# The Status of Hospital Domestic Service

**Published:** 1920-08-14

**Authors:** Gladys M. E. Leigh

**Affiliations:** Nerve Hospital, St. James Road, Edgbaston, Birmingham.


### The Status of Hospital Domestic Service.

*To the Editor of* The Hospital.

Sir,—Your correspondent "A Worker," in her article "The Status of Hospital Domestic Service," quite fails to realise that the women who enter the nursing service of a hospital and those who apply for situations as domestic workers are drawn from two totally different sections of society. The women who enter our nurse-training schools are principally recruited from families whose fathers, brothers, and sisters are engaged in the professions of teaching, medicine, or law, whereas the domestic workers' relatives are usually engaged in manual labour. Many large hospitals now give their cooks the status of sisters and staff nurses, but they are women who have passed through a school of domestic economy and are adequately qualified as trained professional women. Edinburgh Royal Infirmary not only has one principal lady cook, but others who rank as staff nurses. Institutional housekeeping and the care of linen are subjects which are included in the curricula of the training-schools, and every nurse who eventually aspires to an administrative post is aware she must be proficient in these branches of her profession.

In a well-regulated hospital the domestic workers have a sitting-room. The salaries now paid to domestic workers in institutions are excellent. A kitchenmaid devoid of all experience receives higher pay than a nurse in her third year of training, and it is as futile to suggest that domestic service in a hospital should be a stepping-stone into the nursing service as it would be to suggest that a R.A.M.C. orderly might advantageously qualify in medicine.—Yours faithfully, Gladys M. E. Leigh.

Nerve Hospital, St. James Road, Edgbaston, Birmingham.

August 9, 1920.

